# Serum Anticholinergic Activity: A Possible Peripheral Marker of the Anticholinergic Burden in the Central Nervous System in Alzheimer's Disease

**DOI:** 10.1155/2014/459013

**Published:** 2014-02-09

**Authors:** Koji Hori, Kimiko Konishi, Masayuki Tani, Hiroi Tomioka, Ryo Akita, Yuka Kitajima, Mari Aoki, Sachiko Yokoyama, Kazunari Azuma, Daisuke Ikuse, Norihisa Akashi, Misa Hosoi, Koichi Jinbo, Mitsugu Hachisu

**Affiliations:** ^1^Department of Psychiatry, Showa University Northern Yokohama Hospital, Kanagawa 224-8503, Japan; ^2^Tokyo Metropolitan Tobu Medical Center for Persons with Developmental/Multiple Disabilities, Tokyo 136-0075, Japan; ^3^Department of Psychiatry, Showa University Karasuyama Hospital, Tokyo 157-8577, Japan; ^4^Department of Anesthesiology, School of Medicine, Juntendo University, Tokyo 113-8421, Japan; ^5^Department of Clinical Psychopharmacy, School of Pharmaceutical Sciences, Showa University, Tokyo 157-8577, Japan

## Abstract

We review the utility of serum anticholinergic activity (SAA) as a peripheral marker of anticholinergic activity (AA) in the central nervous system (CAA). We hypothesize that the compensatory mechanisms of the cholinergic system do not contribute to SAA if their system is intact and that if central cholinergic system deteriorates alone in conditions such as Alzheimer's disease or Lewy body dementia, CAA and SAA are caused by way of hyperactivity of inflammatory system and SAA is a marker of the anticholinergic burden in CNS. Taking into account the diurnal variations in the plasma levels of corticosteroids, which are thought to affect SAA, it should be measured at noon or just afterward.

## 1. Introduction

Anticholinergic activity (AA) has various adverse effects on both the central nervous system (CNS) and other parts of the body (peripheral tissues) [1]. In CNS, AA causes cognitive disturbances, especially memory disturbances [2–4], executive dysfunction [5], disturbances in diurnal rhythm, and psychotic symptoms such as delusions and hallucinations [6–9]. Although the measurement of AA or anticholinergic burden in CNS is difficult, two methods have been devised. The first method involves the use of a scoring scale using *ex vivo* analysis of AA in various medications. The instruments used for this include the Anticholinergic Drug Scale [10] and Anticholinergic Risk Scale [11]. The other method is the quantification of the serum AA (SAA) using a radioreceptor-binding assay [12]. Rudd et al. commented that an expert-based medications list is the best method for estimation of AA in CNS (CAA) [13]. There are, however, some limitations to this method. First, AA is calculated on the basis of *ex vivo* analysis and there are >600 medications that are thought to have AA. In addition, there are individual differences in pharmacodynamics, pharmacokinetics, and blood-brain barrier permeability between prescription drugs [14].

Moreover, there are interactions among medications and there is a possibility of other condition that causes AA than prescription of medications [15–17]. With regard to SAA, there is a question of the transfer of AA between CNS and peripheral tissues, that is, permeability of the blood-brain barrier. The substances that appear in the serum or in the brain and that are related to positive SAA or CAA have not been identified yet [18]. In this paper, we consider the utility of SAA as a peripheral marker of CAA in view of the relationship between SAA and CAA.

## 2. Utility and Limitations of SAA as a Peripheral Marker of the Anticholinergic Burden in CNS

SAA has been quantified by means of a radioreceptor-binding assay using muscarinic receptors in the forebrains excised from rats. This assay measures inhibition of the binding of radiolabeled quinuclidinyl benzilate, L-[benzilic-4,4′^3^H]quinuclidinyl benzilate ([^3^H]QNB), to rat brain muscarinic acetylcholine (ACh) receptors [12]. Therefore, it is affected by all substances that can bind to muscarinic receptors. As mentioned above, the substances that appear in the serum or in the brain and are related to positive SAA or CAA are not known. Therefore, it is worthwhile to identify these substances, and this task is especially relevant in patients with Alzheimer's disease (AD), in whom the cerebral cholinergic system is thought to be involved in the pathogenesis. Although aberrations in the cholinergic system can involve agonists and antagonists of muscarinic receptors, almost all such aberrations generally have antagonistic actions. Thus, an elevated AA almost always means deterioration of the cholinergic system. Because SAA correlates with AA in the cerebrospinal fluid (CSF) [19, 20] and with the severity of delirium [15, 21–27], SAA can be considered a peripheral marker of CAA.

With respect to cognitive function, patients with schizophrenia, older people (in their own home or nursing home), have been studied to identify possible associations of the anticholinergic burden with cognitive disturbances [2–4, 28–30]. Although the relationship between SAA and MMSE score (minimental state examination [31]; a test of global cognitive function) is not consistent, SAA correlates with memory disturbances especially in demented patients with decreased ACh [2–4]. In studies that have assessed behavioral and psychological symptoms of dementia, SAA correlates with the occurrence of delusions and hallucinations. Moreover, some authors have described the central cholinergic deficiency as characterized by neuropsychiatric symptoms rather than by cognitive dysfunction [6, 7, 9].

Nonetheless, recently, especially in long-term observational studies, SAA was reported to be unrelated to delirium [32–34]. Lampela et al. analyzed data from participants in the population-based Geriatric Multidisciplinary Good Care of the Elderly Study (Kuopio, Finland) and found that SAA is not associated with cognitive dysfunction [34]. Therefore, there are doubts about the association of SAA with AA in CNS.

## 3. Endogenous Emergence of AA in AD

In general, AA in the human body is considered to be mainly a result of prescription drugs, especially those with potent AA and a complex administration regimen [22]. There are, however, reports in the literature that the appearance or a sharp increase in AA can be caused not only by exogenous but also by endogenous factors. Inflammation [15] and stress (cortisol) [16] are thought to be associated with the emergence of AA. Moreover, we have hypothesized that anomalies of the cholinergic system can result in increased AA and reviewed putative mechanism that might cause AA [17, 18].

**Figure 1:**
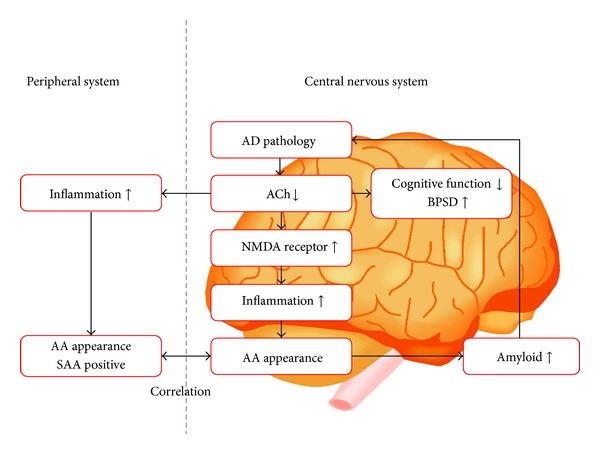
We can speculate that decrease in acetylcholine levels not only causes cognitive dysfunction and behavioral/psychological symptoms of dementia (BPSD) but also allows for inflammatory processes in the central nervous system and in peripheral tissues. The latter changes bring about anticholinergic activity (AA) in both the central nervous system and peripheral tissues by cytokine activation. AA in turn promotes the buildup of amyloid, which further downregulates the cholinergic system. We call this vicious cycle an “endogenous AA cascade.” AA: anticholinergic activity. ACh: acetylcholine. AD: Alzheimer's disease. BPSD: behavioral and psychological symptoms of dementia. NMDA: N-methyl-D-aspartate. SAA: serum anticholinergic activity. This figure is from the article by Hori et al. [18].

In brief, ACh participates not only in cognitive functions but also in inflammatory processes. In AD, where the cholinergic system is downregulated [35], inflammatory processes in both CNS and in peripheral tissues might be caused by the downregulation of ACh. After that, cytokines that have AA might appear as a result of the inflammation. Therefore, we previously hypothesized that AA (both CAA and SAA) might appear endogenously in the moderate stage of AD, where the downregulation of the cholinergic system is substantial; the hyperactivation of N-methyl-D-aspartate (NMDA) receptor expression is upregulated which causes hyperactivity of inflammatory system and AA appears by way of hyperactivity of inflammation; accordingly, we proposed the “endogenous anticholinergic hypothesis in AD” [17, 18] (Figure 1; courtesy Hori et al. [18]). We also reported the case of a 76-year-old man with AD in the moderate stage whose SAA became positive when his memory disturbances, disorientation, apathy, and aphasia had worsened [36]. His SAA disappeared after 3 months of treatment with memantine, an antidementia agent that is an NMDA receptor antagonist. At the same time, his apathy and aphasia were ameliorated. Because downregulation of the cholinergic system is an essential feature in AD [35], we hypothesized that AA might appear endogenously in the moderate stage of AD. Alternatively, if the cholinergic system is not downregulated, AA should not appear.

## 4. The Compensatory Mechanism of the Cholinergic System Does Not Give Rise to AA

We also reported the case of a 74-year-old woman who presented with amnesia and positive SAA at the stage of mild cognitive impairment, which disappeared after treatment with the cholinesterase inhibitor donepezil for 1 year [37]. We believe that both mental stress and AD caused SAA in this patient. It is also possible that SAA may have subsequently become negative (undetectable) because of downregulation of inflammation by way of upregulation of ACh production by donepezil. Because the patient's other medications and physical condition remained unchanged during the 1-year period, we assumed that the appearance of SAA was because of proinflammatory changes caused by both ACh deficiency and psychological stress [37]; in other words, the concurrence of AD (cholinergic system downregulation) and psychological stress caused substantial inflammatory activities, which produced AA. We arrived at this supposition on the basis of a report that compensatory increase in choline acetyltransferase activity occurs in patients with mild cognitive impairment and early AD [38, 39]. Moreover, it has also been reported that ACh release can be enhanced in the hippocampus and prefrontal cortex by exogenous stress [40, 41]. Therefore, we supposed that this compensatory reaction might be present not only in mild cognitive impairment and early AD but also in psychological stress [37].

On the basis of the above observations, we hypothesized that if the cholinergic system is intact, AA does not emerge because upregulation of the cholinergic system inhibits the appearance of AA by way of suppression of inflammatory processes. Even if SAA appeared (e.g., by prescription medication or due to inflammation in the peripheral tissues), the intact central cholinergic system can be activated and can compensate for peripheral inflammation, even though peripheral AA promotes inflammation. Therefore, in this healthy state, inflammation in CNS and central AA cannot happen.

**Figure 2:**
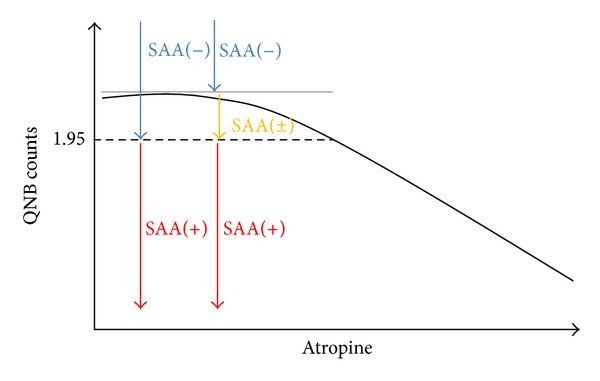
The relationship between atropine concentration (the amount of atropine in a standard solution: given in nM) and [^3^H]QNB counts is linear from 1.95 nM to 25 nM. Generally, SAA is positive if [^3^H]QNB counts are under the level corresponding to 1.95 nM; therefore, SAA is considered positive [SAA(+)] when it is ≥1.95 nM (left red arrow); SAA is undetectable [SAA(−)] when it is <1.95 nM (left blue arrow). Nevertheless, because a small amount of SAA can cause AA in the central nervous system, clinicians should keep in mind that SAA might also be positive if [^3^H]QNB counts are above the level corresponding to 1.95 nM (0 nM < SAA < 1.95 nM; right yellow arrow). [^3^H]QNB: tritiated quinuclidinyl benzilate, SAA: serum anticholinergic activity.

On the other hand, if the central cholinergic system has deteriorated, then it cannot be activated and cannot compensate for the peripheral activity of inflammatory processes. In this case, inflammation in CNS arises, and CAA increases. Accordingly, we hypothesized that SAA might be a marker of the anticholinergic burden in CNS only when the central cholinergic system has deteriorated, similar to the state in AD or Lewy body dementia. As mentioned above, SAA and CAA are not well understood and neither is the ability of AA to cross the blood-brain barrier. Nonetheless, research shows that SAA correlates with AA in CSF [19, 20]. Tune et al. [26] reported that postoperative SAA of ≥7.5 nM in patients without dementia is associated with a higher risk of delirium. On the other hand, Tune and Coyle [12] showed that SAA of ≥3.5 nM is necessary for beneficial effects of antipsychotics in schizophrenic patients in order to avoid extrapyramidal side effects; these data are suggestive of a delicate balance between dopaminergic and cholinergic neuronal activities. Generally, any SAA level greater than the detection of limit of a quantitative level (≥1.95 nM in our study) is defined as positive SAA [SAA(+)]. According to previous studies SAA is considered to be positive when it is ≥1.95 nM and undetectable when it is <1.95 nM. Nevertheless, because a small amount of SAA can cause AA in CNS, we should assume that SAA might also be positive if its concentration is between 0 nM and 1.95 nM (0 nM < and <1.95 nM; Figure 2).

We previously investigated the relationship between SAA and the severity of symptoms of AD [4, 9]. Twenty-six out of 76 patients with AD referred to our hospital were SAA(+), and the remaining 50 patients were negative [SAA(−)]. The SAA(+) group showed significantly greater global cognitive dysfunction and higher scores on the items of paranoid and delusional ideation, hallucinations, and diurnal rhythm disturbances. The average concentration of SAA in the SAA(+) group was 4.14 ± 2.70 nM (mean ± SD) [4, 9]. This concentration is lower than the postoperative cut-off level for delirium in patients without dementia, and somewhat higher than the lower limit necessary to avoid extrapyramidal side effects in patients with schizophrenia, as reported by Tune and Coyle [12]. In addition, we reported that SAA of 2.38 nM caused adverse effects on cognitive function in a patient with AD [36]. However, we thought that it was quite probable that even low values of SAA could affect cognition and behavioral/psychological symptoms of dementia in AD, albeit with no effect on cognition in patients without dementia [2, 3]. AA easily worsens AD symptoms. We now, however, believe that because in AD the cholinergic system has deteriorated, a small amount of SAA might cause increases in CAA, sufficient to produce adverse effects on cognitive function and psychotic symptoms. On the contrary, a much higher amount of SAA might be needed for patients without dementia in order to trigger increased CAA.

In summary, there are associations between SAA and AA in CSF. It means not that AA in CSF and SAA influence each other through the blood-brain barrier but that the downregulation of cholinergic system allows for activation of inflammation in both CNS and the peripheral tissues. The latter changes bring about increased CAA and SAA through cytokine activation. Therefore, there are mechanistic links between SAA and AA in CSF when the CNS cholinergic system has deteriorated.

As mentioned above, SAA assays were originally developed as a receptor-binding assay for muscarinic receptors in the forebrains excised from rats, and this assay measures inhibition of [^3^H]QNB counts (tritiated quinuclidinyl benzilate counts: given in disintegrations per minute) associated with rat brain muscarinic ACh receptors [12]. The relationship between atropine concentration (the amount of atropine in a standard solution: given in nM) and [^3^H]QNB counts is linear from 1.95 nM to 25 nM. The detection limit of SAA is approximately 1.95 nM (Figure 2), and patients with AD who might have been affected by lower SAA could be incorrectly assumed to be SAA(−). However, further research is needed on the endogenous appearance of AA in AD.

## 5. When Should We Measure SAA?

The next important item on the agenda is when SAA should be measured. If inflammatory processes cause AA, it is natural to think that steroids should inhibit AA because of their anti-inflammatory properties. On the other hand, steroids have been reported to probably induce or increase AA [16]. In fact, depression-like behavior caused by adrenocorticotrophic hormone in rats is ameliorated by the N-methyl-D-aspartate (NMDA) receptor antagonist memantine [42]. Moreover, Takahashi et al. reported that corticosterone acutely activates NMDA receptor in the rat hippocampus [43].

**Figure 3:**
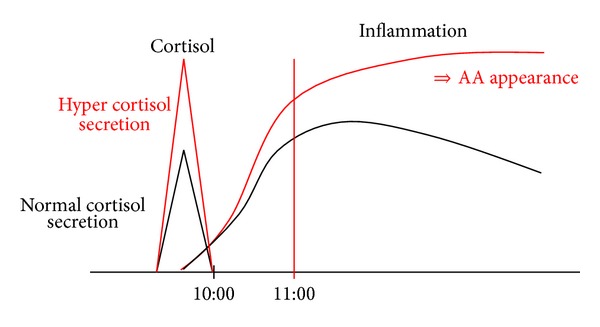
The plasma steroid level is high early in the morning and rapidly declines later. We hypothesized that this rapid decline might cause disinhibition of the immune system. Thus, the immune system may be activated in the afternoon, in the evening, and at night. If the levels of steroids in blood rise, then the decline of the steroid level also becomes larger, which causes a more active inflammatory state and increased anticholinergic activity (AA, red arrow). AA: anticholinergic activity.

The corticosteroid level in plasma is high early in the morning and rapidly declines after that [44–46]. We theorized that this rapid decline might cause disinhibition of the immune system; thus, the immune system may be activated in the afternoon, in the evening, and at night. If the blood level of steroids rises above usual values, the decline of the steroid levels should also become larger, which is expected to cause a more active inflammatory state and AA (Figure 3). Therefore, it seems logical that even if AA does not appear early in the morning, it may appear at noon or somewhat later. This mechanism might explain why even if a patient with delirium seems calm in the mornings, he becomes delirious in the late afternoon and at night. Therefore, SAA is best measured at noon or somewhat later. These two topics, that is, the diurnal rhythm of AA and the relationship between AA and corticosteroids, need more research.

## 6. Clinical Implications

On the basis of the above theories, three main putative causes of endogenous appearance of AA, that is, fabric illnesses [15], stress [16], and downregulation of ACh [17], all might be associated with increased activity of inflammatory pathways. The inflammatory processes and AA might be a final common pathway in the pathogenesis of AD (formation of amyloid plaques). More studies are needed to elucidate whether AA appears endogenously in AD because of inflammation. In any case, when evaluating SAA in AD, we believe that an attempt should be made at minimizing AA in patients with AD. Physicians and family members should protect these patients from physical illnesses and mental stress in order to not worsen cognitive deficits. When clinicians see patients with AD who are having psychotic and other symptoms (e.g., delusions, hallucinations, and disturbances in diurnal rhythm), they should check prescribed medications, physical illnesses, and mental stress because these factors can affect the cholinergic system and thereby contribute to psychosis. If these factors are not to blame, clinicians should firstly consider nonpharmacological modalities. If these methods are not effective, psychotropic medications without AA should be considered.

It appears that downregulation of cholinergic system should be avoided in AD because it may cause not only cognitive dysfunction but also acceleration of AD. Therefore, it is often necessary to prescribe cholinergic antidementia medication if the diagnosis of AD is made. Moreover, if AA (both CAA and SAA) might appear endogenously in the moderate stage of AD, SAA might be a biological marker for prescribing memantine; that is, if SAA is positive we should prescribe memantine.

## 7. Conclusions

We reviewed the possible causes of the endogenous appearance of AA in the moderate stage of AD. From the case report of a patient at the stage of mild cognitive impairment, who presented with amnesia and positive SAA, which disappeared after treatment with a cholinesterase inhibitor, we hypothesized that AA is suppressed by the compensatory mechanism of the cholinergic system. This mechanism does not generate and actually prevent AA; thus, when the cholinergic system has deteriorated, the compensatory mechanism does not work, and consequently, the anticholinergic burden in CNS might appear. According to the above theory, SAA may serve as a marker of anticholinergic burden in the brain only when the central cholinergic system has deteriorated, as is the case in AD or Lewy body dementia. Because of diurnal variation of the plasma steroid levels, it is best to measure SAA at noon or somewhat afterward.
